# Inhibition of Histone Deacetylase Activity in Human Endometrial Stromal Cells Promotes Extracellular Matrix Remodelling and Limits Embryo Invasion

**DOI:** 10.1371/journal.pone.0030508

**Published:** 2012-01-26

**Authors:** Carlos Estella, Isabel Herrer, Stuart P. Atkinson, Alicia Quiñonero, Sebastián Martínez, Antonio Pellicer, Carlos Simón

**Affiliations:** 1 Fundación Instituto Valenciano de Infertilidad (FIVI), Valencia University, and Instituto Universitario IVI/INCLIVA, Valencia, Spain; 2 Centro de Investigación Príncipe Felipe, Valencia, Spain; State Key Laboratory of Reproductive Biology, Institute of Zoology, Chinese Academy of Sciences, China

## Abstract

Invasion of the trophoblast into the maternal decidua is regulated by both the trophoectoderm and the endometrial stroma, and entails the action of tissue remodeling enzymes. Trophoblast invasion requires the action of metalloproteinases (MMPs) to degrade extracellular matrix (ECM) proteins and in turn, decidual cells express tissue inhibitors of MMPs (TIMPs). The balance between these promoting and restraining factors is a key event for the successful outcome of pregnancy. Gene expression is post-transcriptionally regulated by histone deacetylases (HDACs) that unpacks condensed chromatin activating gene expression. In this study we analyze the effect of histone acetylation on the expression of tissue remodeling enzymes and activity of human endometrial stromal cells (hESCs) related to trophoblast invasion control. Treatment of hESCs with the HDAC inhibitor trichostatin A (TSA) increased the expression of TIMP-1 and TIMP-3 while decreased MMP-2, MMP-9 and uPA and have an inhibitory effect on trophoblast invasion. Moreover, histone acetylation is detected at the promoters of TIMP-1 and TIMP-3 genes in TSA-treated. In addition, in an *in vitro* decidualized hESCs model, the increase of TIMP-1 and TIMP-3 expression is associated with histone acetylation at the promoters of these genes. Our results demonstrate that histone acetylation disrupt the balance of ECM modulators provoking a restrain of trophoblast invasion. These findings are important as an epigenetic mechanism that can be used to control trophoblast invasion.

## Introduction

Implantation of the human embryo in the maternal endometrium is a key step for the successful establishment of pregnancy and requires dialogue between the competent embryo and the receptive endometrium [1]. Stromal decidualization is a critical process that allows correct trophoblast invasion and placenta formation [2]. This process includes morphological and biochemical changes of the fibroblast-like stromal cells by the action of ovarian steroids 17β-estradiol (E_2_) and progesterone (P_4_). A network of signalling molecules and transcription factors which controls the decidualization process has been identified [3], [4].

Invasion of the trophoblast into the maternal decidua is regulated by both the trophoectoderm and the stroma, and requires the action of tissue remodelling enzymes [5]. Metalloproteinases (MMPs) are a family of zinc-dependent enzymes that play a key role in degrading components of the extracellular matrix (ECM). These enzymes regulate multiple physiological events such as cell differentiation, cell motility, inflammation and, in disease, tumour progression [6]. One of the major constituents of decidual ECM is collagen IV, which is a substrate of the 72-kDa and 92-kDa type IV collagenases, MMP-2 and MMP-9, respectively [7]. Both MMPs are expressed and secreted by the human endometrium and there is evidence that their function is required for the tissue remodelling process during implantation or for menstruation to occur in the absence of pregnancy [8], [9], [10]. Trophoblast invasion, like tumour invasion, requires the active secretion of these proteases, which are capable of digesting the ECM of the endometrium [11]. In contrast, decidual stromal cells activate the expression of the tissue inhibitors of MMPs (TIMPs) and down-regulate the urokinase plasminogen activator (uPA) [9]. It has been proposed that TIMPs act to limit ECM degradation by MMPs in the endometrium during decidualization, thus limiting embryonic invasion. The balance between these promoting and restraining factors is crucial for the successful outcome of pregnancy [12], [13]. Aberrant expression or distribution of ECM components in the decidual stroma has been associated with preeclampsia or restricted intrauterine growth [14], [15], and an abnormal balance between MMPs and TIMPs has been related to tumour invasion and metastasis in various human cancers, including endometrial cancers [16], [17].

An increasing body of evidence indicates that changes in chromatin structure by histone modification appear to play an important role in the regulation of gene transcription. Histone modifications are very dynamic and reversible during normal development and their misregulation is associated with cancer [18]. Histone acetylation, one of the best characterized histone modifications, unpacks condensed chromatin facilitating the access of transcription factors to target gene promoters. Histone deacetylases (HDACs), along with histone acetyl transferases (HATs), regulate the acetylation of histones and HDAC inhibition can induce gene expression.

During the menstrual cycle, class I HDAC have been shown to be constitutively expressed while global acetylation increases in the early proliferative and secretory phases [19], [20]. Moreover, it has been reported that endometrial stromal cells cultured *in vitro* with E_2_ and P_4_ increase H3 and H4 acetylation [21]. TSA is a potent and specific organic compound that selectively inhibits HDAC families of enzymes and in human endometrial stromal cells, TSA treatment increases the levels of acetylated H3 and H4 [21].

The aim of the present study was to analyze the effect of histone deacetylase inhibition in human endometrial stromal cells (hESCs) and its impact on trophoblast outgrowth and invasion. Our results demonstrate a dysregulation of the TIMPs and MMPs balance due to induction of histone acetylation at the TIMP-1 and TIMP-3 gene promoters. A similar effect was identified during hESCs *in vitro* decidualization through TIMP-1 and TIMP-3 expression induction due to histone acetylation at the TIMP-1 and TIMP-3 gene promoters.

## Material and Methods

### Ethics statement

This study was approved by the Institutional Review Board and Ethics Committee of Instituto Universitario-Instituto Valenciano de Infertilidad (Universidad de Valencia, Spain) (0901-EN-023-FD code), and informed written consent was obtained from each patient prior to tissue collection.

### Endometrial stromal and epithelial cell isolation, cell culture and TSA treatment

Endometrial samples, acquired after obtaining written consent from patients, were collected on the day of oocyte retrieval from healthy ovum donors aged 19–35 years old (n = 25). Biopsies were subjected to mild collagenase digestion to isolate human endometrial stromal cells (hESCs) and human endometrial epithelial cells (hEECs), as previously described [22]. The purity of the cultures was assessed by vimentin (hESCs+, hEECs−) and E-cadherin staining (hESCs−, hEECs+). hESCs were cultured in DMEM/F12 medium containing 10% charcoal stripped foetal bovine serum (FBS) and 0.1% antibiotics. Isolated hEECs were cultured in 75% DMEM and 25% MCDB-105 (Sigma, St. Luis, MO.) containing antibiotics and supplemented with 10% FBS and 5 µg/ml insulin. JEG-3 is an immortalized cell line derived from human placental choriocarcinoma obtained from the American Type Culture Collection (ATCC) (HTB-36; Manassas, VA). JEG-3 was cultured in EMEM (Gibco, Invitrogen) medium containing 10% FBS and gentamicin (50 mg/ml) and fungizone (0.25 mg/ml).

Human foreskin fibroblast cell line (hFFCs) was obtained from the ATCC (CRL-2429; Manassas, VA) and was cultured in IMDM medium supplemented with 10% FBS and 1% antibiotics. HDAC inhibition was carried out with Trichostatin A (TSA) (Sigma) at two different concentrations: 0.1 µM and 1 µM. hESCs were cultured for 48 h with the drug treatment or vehicle (controls) before any analysis. Cell viability was determined by Trypan blue exclusion.

Decidualization was induced by culturing confluent hESCs in DMEM/F12 medium containing 2% FBS with 0.5 mM 8-Br-cAMP and 1 µM medroxyprogesterone (MPA) (Sigma) for 5 days as previously described [23]. The medium was removed every 3 days. The hESCs decidualization phenotype was confirmed by prolactin secretion in conditioned media by Elisa (Abbott Axsym System, Abbot Park, IL).

hESC were treated with either 1 µM TSA for 48 h or 0.5 mM 8-Br-cAMP and 1 µM MPA for 5 days. Then cells were washed with PBS and fresh DMEM-F12 medium containing 2% FSB was added. The conditioned media were collected after 24 h and were subjected to Western blot analyses.

### Wound healing and invasion assays

Motility of hESCs after treatment with TSA was assessed using the wound-healing assay. Briefly, hESCs were grown to confluence and treated with 0.1 µM and 1 µM TSA or vehicle. After 48 h, each well was scratched with a sterile pipette tip, the medium was removed, cells were washed with PBS and cultured in fresh medium. The width of the denuded area was subsequently measured at 0 h and 24 h. Wound closure was calculated as the percentage of the closed area of the initial wound width.

Invasion assays of hESCs were conducted with the Collagen Transwell Invasion kits (Chemicon Int. Billerica, MA). The hESCs previously treated with TSA or vehicle for 48 h were resuspended in DMEM/F12 containing 5% BSA and 2.5×10^5^ cells were plated into 8-µm-pore size transwell inserts while DMEM/F12 medium supplemented with 10% FBS was plated on the bottom as a chemoattractant. Cells were allowed to invade for 48 h. Invasion was measured by OD using a standard microplate reader.

**Figure 1:**
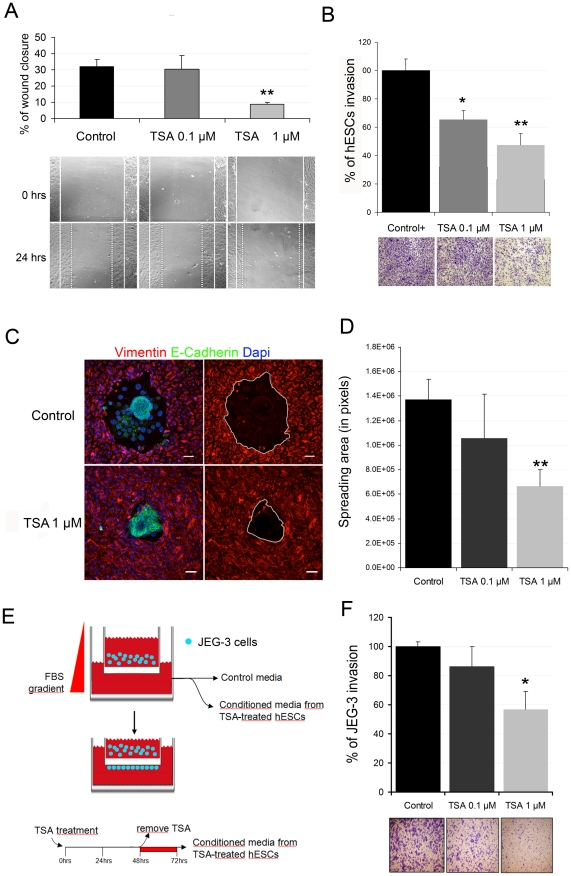
TSA reduces hESC motility and limits trophoblast outgrowth and invasion. **A**, Wound healing assay was performed in 48 hour-treated hESCs at two different TSA concentrations (0.1 µM and 1 µM) or a vehicle (control). The % of migrating cells was calculated as the area covered by cells and is represented as an average of three independent experiments. Statistical analysis ** p<0.01. **B,** Invasion assays of the TSA-treated hESCs were conducted in collagen transwells. hESCs were allowed to invade for 48 h after being previously treated with a vehicle (control), or with 0.1 µM and 1 µM TSA. The histograms show a percentage of invading cells, with the invasion of the control cells designated as 100%. Representative bright field images of the cells invading the underside of the filter are shown below the histograms. Data represent the mean of three independent experiments. Statistical analysis,* p<0.05 and ** p<0.01. **C**, TSA-treated hESCs limit mouse embryos invasion potential. hESCs and the mouse embryo were marked by Vimentin (red) and E-Cadherin (green), respectively. Nuclei were marked by DAPI (blue). The invasion area was identified by the absence of stromal cells (red) and outlined by a white line. Bar size is 50 µm. **D**, 1 µM TSA-treated hESCs significantly decreased the area of embryo spreading. Data represent the mean areas of at least 8 embryos in three independent experiments. Statistical analysis, ** p<0.01. **E,** Schematic representation of the collagen transwell invasion assay used to measure the effect of the TSA-treated hESCs conditioned medium on human trophoblast cells. See Material and Methods for procedure description. **F,** Histograms show the percentage of the invading cells, with invasion of the control cells designated as 100%. Representative bright field images are shown below the histograms. Representative bright field images of the cells invading the underside of the filter are shown below the histograms. Data represent the mean of three independent experiments.

The invasion assay of cytotrophoblastic cell line JEG-3 [24] was measured using the collagen-invasion chamber (see Figure 1E). hESCs were treated for 48 h with 0.1 µM and 1 µM of TSA or vehicle, the medium was replaced, cells were washed with PBS and fresh medium was added for 24 h to obtain the conditioned media. In the upper chamber, 2.5×10 ^5^ JEG-3 cells were resuspended in the 24 hour-hESC conditioned medium and plated into 8-mm-pore size transwell inserts. JEG-3 cells were allowed to invade for 48 h and invasion was measured by OD using a standard microplate reader.

### Gelatin Zymography

Twenty-four-hour serum-free conditioned media were collected after 48 h TSA-treated and control hESC and were mixed with a non-reducing sample buffer. Seven µg of protein per well were separated by 10% polyacrylamide gels containing 1 mg/ml gelatine. After electrophoresis, MMPs were renatured by washing the gel in 50 mM Tris-HCl, pH 8, containing 2.5% Triton X-100 for 1 h at room temperature. MMPs were then activated by incubating the gel at 37°C overnight in 50 mM Tris-HCl, pH 8, containing 5 mM CaCl_2_ as previously described [25]. The gel was stained with 0.1% (w/v) Coomasie Brilliant blue R-250 and destained in distilled water/glacial acetic acid/methanol (4.5∶1∶4.5) (v/v). Band densitometries were analyzed using the Image J software to measure peak areas (http://rsbweb.nih.gov/ij/links.html).

### Embryo Collection, Co-Culture and Immunohistochemistry

Procedures performed on animals were approved by the Institutional Review Board and Ethics Committee of Instituto Universitario-Instituto Valenciano de Infertilidad (Universidad de Valencia, Spain). B6CBF1 strain mice were superovulated and mated with males of the same strain. Two-cell embryos were recovered on 2 day postcoitum and cultured for another 2-day period in CCM™ medium (Vitrolife). Hatched embryos were added to the confluent monolayers of hESCs treated 48 h previously with TSA or vehicle. Co-cultures were fixed in 4% paraformaldehyde in PBS with 0.1% Triton and 0.1% sodium deoxycholate for 25 minutes. Cells were then blocked with PBS 0.3% Triton, 0.03% sodium azide and 1% BSA for 30 minutes. Cells were incubated with primary antibodies (vimentin and E-cadherin) in the same blocking solution buffer overnight at 4°C, followed by the appropriate secondary conjugated antibody (Molecular probes) for 1 h at room temperature. Cells were mounted in Vectashield mounting medium containing DAPI (Vector Laboratories). Trophoblast invasion was analysed 48 h after embryo co-culture by measuring the spreading of the embryo in the hESCs by immunofluorescence using the vimentin (Sigma) and E-cadherin (Abcam Cambridge, UK) antibodies. Trophoblast spreading was quantified by measuring the wound area in the stromal cells made by the invading embryo using the Image J software. A total of 8 embryos were used for the experiment and each experiment was done in hESCs isolated from three different biopsies.

### RT and quantitative real-time PCR analysis

Total RNA was extracted from cell cultures using TRIzol reagent (Invitrogen) following the manufacturer's protocol. Firstly, 1 µg was reverse-transcribed with the M-MLV RT system (Promega) to generate cDNA for the quantitative and semi-quantitative analyses. Quantitative real-time PCR was performed in triplicate using LightCycler FastStart DNA Master SYBR green I (Roche) in a LightCycler 480 (Roche). The mRNA level was normalized to GAPDH as a house keeping gene. The primers sequences used were: MMP-2, 5′-CAAAAACAAGAAGACATACATCTT-3′and 5′-GCTTCCAAACTTCACGCTC-3′; MMP-9, 5′-TGGGGGGCAACTCGGC-3′and 5′-GGAATGATCTAAGCCCAG-3′; TIMP-1, 5′-CTG TTG TTG CTG TGG CTG ATA-3′ and 5′-CCG TCC ACA AGC AAT GAG T-3′; TIMP-3, 5′-ATGGTGTAGACCAGCGTGC-3′and 5′-AGGACGCCTTCTGCAACTC-3′; uPA, 5′-CACGCAAGGGGAGATGAA-3′and 5′-ACAGCATTTTGGTGGTGACTT-3′; RECK, 5′-CTCGGTTTGTTGCAGTTATGC-3′and 5′-AGAGGCGCAATAATTTTCCACT-3′; GAPDH, 5′-GAAGGTGAAGGTCGGAGTC-3′and 5′-GAAGATGGTGATGGGATTTC-3′.

### Western Blots

Conditioned media (CM) was obtained as previously described. The proteins from the CM were precipitated with acetone at −20°C for at least 3 h and were centrifuged at 6000 g. Pellets were resuspended in lysis buffer (300 mM NaCl, 20 mM Tris, 10 mM EDTA, 2% (v/v) Triton X-100, pH = 7.3) and supplemented with a protease inhibitor cocktail.

The proteins (40 µg/lane) from the CM were separated on 10% SDS-polyacrylamide gels, transferred to polyvinylidene difluoride membranes (BIO-RAD) and blotted overnight at 4°C with antibodies specific to human TIMP-1 and TIMP-3 (Abcam) at 1∶250 and 1∶1000 dilutions, respectively. After washing three times, blots were incubated with diluted horseradish peroxidase-conjugated second antibodies (Santa Cruz Biotechnology, St. Cruz, CA) for 1 h at room temperature. Blots were then washed extensively and developed using enhanced chemiluminescence. Ponceau membrane staining was used for data normalization.

### Chromatin Immunoprecipitation Assay (ChIP) and PCR

ChIP was undertaken following the instructions recommended by the kit supplier (Millipore, Cat no. 17-295). Briefly, hESCs were treated for 24 h with 1 µM TSA or with 0.5 mM 8-Br-cAMP and 1 µM MPA, washed with PBS, fixed using 1% formaldehyde and lysed. Then chromatin was sonicated to give chromatin fragments of around 500 bp in length. Chromatin was then immunoprecipitated using ChIP Grade antibodies against acetylated Histone H3 (Millipore, 06-599), acetylated Histone H4 (Millipore, 06-598) and a nonspecific IgG-control antibody. Sodium butyrate was used to inhibit DNA de-acetylation throughout the lysis and incubation steps. Subsequently, immunoprecipitated DNA was washed, eluted and then purified using ethanol precipitation and a Qiaquick DNA Purification kit (Qiagen, West Sussex, UK) prior to the Q-PCR analysis. Q-PCR was undertaken using the SYBR Green QPCR mastermix (Sigma, Paisley, UK) in a Roche LC480 LightCycler. These values were compared to an input control sample which represents a 10% fraction of the total chromatin used in each IP.

Primers were designed to amplify a region in the 5′ regulatory regions of TIMP-1 and TIMP-3 near the transcription start site, and promoter regions and CpG islands were suggested whenever possible using WebPromoterScan (http://wye.cit.nih.gov/molbio/proscan/).

Primers: TIMP-1 5′-TTTCCTCTCTGCCACCCCTCACCA-3′ and 5′-CCGCCTCAGCGGAGAGCTTAG-3′; TIMP-3 5′-CGGCAATGACCCCTTGGCTCG-3′ and 5′-GGAGCGCTTACCGATGTCGGA-3′.

A non antibody control immunoprecipitate was also included in each ChIP experiment to detect any background, if present, as detected by qPCR, and was subtracted from the values found for each immunoprecipitate in that experiment.

### Statistics

At least three different biopsies were used per experiment and measurements were taken in triplicate. Data are presented as the mean and the standard error mean (SEM), and the statistical significance of differences were evaluated with the Student's t-test (* p<0.05 and ** p<0.001).

## Results

### TSA reduces hESC motility and limits trophoblast outgrowth and invasion

The spreading and invasion of the embryonic trophoblast require the motility of maternal hESCs [26], [27], [28]. To explore the effect of HDCA inhibitor TSA on stromal cell motility, we used two different concentrations of TSA (0.1 µM and 1 µM) which did not significantly affect the viability of cells at 48 h post-treatment in a cell survival assay (Figure S1A). TSA at the highest concentration (1 µM) significantly reduced cell migration (from 32% in control to 8% in TSA-treated hESCs, 75% compared to control) in the *in vitro* wound-healing assay, while the lowest concentration (0.1 µM) did not affect it (Figure 1A).

The effect of TSA on the ability of hESCs to move through a collagen barrier was examined by the collagen-invasion chamber assay. The percentage of endometrial stromal cells invading the collagen layer compared to the positive control significantly reduced in a dose-dependent manner (Figure 1B). hESCs treated with 0.1 µM and 1 µM TSA were respectively 38% and 57% less motile through the collagen layer than control cells. These findings suggest that TSA reduces the motility capacities of hESCs.

We also used a heterologous co-culture *in vitro* model to investigate the outgrowth and invasion of mouse embryos in TSA-treated hESCs. The blastocysts placed on a confluent monolayer of hESCs attached to them 24∼48 h later [29]. This *in vitro* model has been successfully used to study the role of Rho GTPase RAC1 in the motility of hESCs and, hence, embryo invasion [27], [28]. We used hESCs pre-treated with TSA for 48 h before embryo co-culture to avoid any possible side effects of TSA on the embryo. Hatched blastocysts were added to confluent TSA pre-treated stromal cells, and the co-culture was fixed 48 h later and stained for E-cadherin and vimentin as markers for trophoblast and stromal cells, respectively. The attached embryos in the control hESCs exhibited extended trophoblast spreading, while trophoblast outgrowth in 1 µM TSA pre-treated cells reduced by 50% if compared to control cells (Figure 1C and 1D).

The effect of TSA-treated stromal cell on human trophoblast cell invasion was studied by the collagen-invasion chamber assay. The invasive potential of the JEG-3 cell line cultured in conditioned media from 48 hour-TSA-treated stromal cells (0.1 or 1 µM) was measured (Figure 1E, and Material and Methods). Invasion of the JEG-3 cells was inhibited by 42% in response to 1 µM TSA-treated hESCs conditioned medium if compared to the vehicle (Figure 1F). Our results suggest that histone deacetylation inhibition in hESCs restrain trophoblast invasion and outgrowth.

### TSA induced an alteration in the balance of TIMPs and MMPs in hESCs

**Figure 2:**
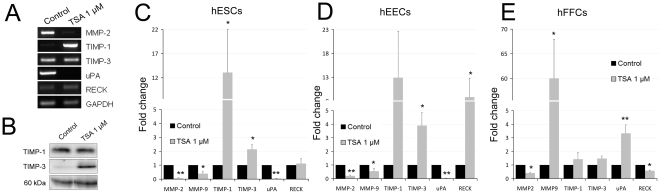
TSA alters the expression of the ECM modulating enzymes. **A,** Semi-quantitative PCR of ECM modulators MMP-2, TIMP-1, TIMP-3, uPA and RECK in 48 h 1 µM TSA hESCs. Note that MMP-9 is not shown because the low expression levels were barely detectable in agarose gel. **B**, Extracellular TIMP-1 and TIMP-3 Western blot analyses of protein extracts of 24 hour-conditioned media from 48 hour-hESCs treated with 1 µM TSA. Ponceau staining of membranes was used as a protein loading control. Only the 60 kDa band of the ponceau gel is shown. Representative image of three different analyses is shown. **C**, **D** and **E**, Quantitative PCRs for the MMP-2, MMP-9, TIMP-1, TIMP-3, uPA and RECK expression levels in human endometrial stromal cells (hESCs, B), endometrial epithelial cells (hEECs, C) and foreskin fibroblast cells (hFFCs, D). Data represent the mean of three independent experiments. Statistical analysis,* p<0.05 and ** p<0.01.

Inhibition of histone deacetylases with 1 µM TSA in hESCs significantly increased the expression of TIMP-1 (13-fold), and of TIMP-3 (2.1-fold) at the mRNA level, while it decreased the MMP2 (−14.7-fold) and MMP9 (−2.5-fold) levels analyzed by semi-quantitative and quantitative PCR (Figure 2A and 2C). Moreover, the levels of urokinase plasminogen activator (uPA) were significantly reduced (−23.2-fold). This protease is responsible for ECM remodelling during menses, and plays a role in tumour invasion and metastasis [9], [30]. In contrast, another MMP regulator, the tumour suppressor RECK [31], did not alter at the mRNA level in the TSA-treated cells if compared to controls. At the protein level, TIMP-3 was up-regulated in 24-hour conditioned media from hESCs previously treated for 48 h with 1 µM TSA or vehicle (Figure 2B). In contrast, we did not observe increased TIMP-1 protein levels in the same conditioned media, although these cells showed an increase at the mRNA level of TIMP-1 (Figure 2B). Altogether, these results indicate that TSA-treated hESCs lower the expression of the ECM proteases enzymes, while they promote the expression of protease inhibitors.

To determine whether this is a specific effect on hESC, we studied the effect of this drug on human endometrial epithelial cells (hEECs) and human foreskin fibroblast cells (hFFCs). A quantitative PCR analysis demonstrated that hEECs treated with 1 µM TSA behave similarly to hESCs with reduced MMP-2 (−4.6-fold), MMP-9 (−2-fold, although not significant) and uPA (−23-fold) mRNA levels, while they display an up-regulation of TIMP-1 (13-fold), TIMP-3 (3.9-fold) and RECK (8.7-fold) expression (Figure 2D). Instead, hFFCs respond differently to the TSA stimulus: MMP-2 decreased (−2.5-fold), MMP-9 increased (60-fold), TIMP-1 and TIMP-3 did not significantly change, while uPA was up-regulated (3.3-fold) and RECK was down-regulated (−1.72-fold) (Figure 2E).

These results suggest that the TSA effect pattern at the MMPs and TIMPs mRNAs level is specific to both stroma and epithelial endometrial cells.

### TSA modulates the active *versus* latent forms of MMP-2 and MMP-9 in hESCs

**Figure 3:**
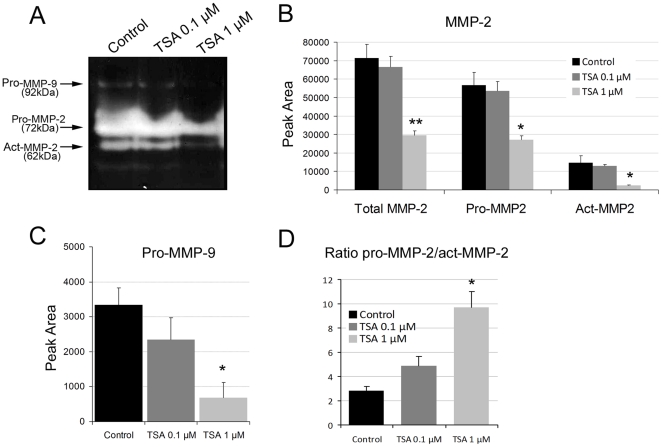
Enzymatic activities of MMP2 and MMP-9 in TSA-treated hESCs. **A,** 24 hour-conditioned serum-free media collected from 48 hour–TSA-treated and control hESC were subjected to gelatin zymography to assess the activation state of MMPs. Three clearly distinct bands of gelatinase activity were observed at 92 kDa (pro-MMP-9), 72 kDa (pro-MMP-2) and 62 kDa (act-MMP-2). It is noteworthy that the 86 kDa band, which corresponds to the active form of MMP-9, was not observed. The image is representative of three independent experiments. **B** and **C**, Quantification of the gelatin zymography results by densitometry of the MMP-2 bands shows a decrease in total MMP-2 and a decrease in the latent (pro-MMP-2) and active forms (act-MMP-2) in TSA-hESCs treated cells. **C,** The latent form of MMP-9 (pro-MMP-9) also decreases in TSA-treated hESCs. **D**, The ratio between the active and the latent forms of MMP-2 increases in TSA hESCs. Data represent the mean of three independent experiments. Statistical analysis,* p<0.05 and ** p<0.01.

To elucidate the molecular differences underlying the TSA-treated hESCs responsible for the limited invasion potential of trophoblast cells, a gelatine zymographic analysis of the supernatant media from the TSA-treated stromal cells was performed to assess the MMP-2 and MMP-9 protein levels and enzymatic activities. Gelatin zymographs resolve gelatinases by their molecular mass and permit a distinction to be made between the latent (pro-MMPs) and active forms (act-MMPs) of MMPs. In the control hESCs, we identified three clearly distinct bands of gelatinase activity at 92 kDa (pro-MMP-9), 72 kDa (pro-MMP-2) and 62 kDa (act-MMP-2), as previously described (Figure 3A). It is noteworthy that the active form of MMP-9, was not observed. We identified a decrease in not only the total amounts of MMP-2 (57%), in both the active and latent forms, but also in pro-MMP-9 (80%) in the hESCs treated with TSA if compared to controls (Figure 3A–3C). The increased ratio between the latent and active forms of MMP-2 (pro-MMP-2/active-MMP-2, 2.8 in controls, 4.8 in 0.1 µM TSA and 9.7 in 1 µM TSA) in the TSA-treated hESCs, if compared to control cells, indicates that TSA reduces MMP-2 activity not only levels, probably due to an increase in one of its inhibitors (TIMP-1 and/or TIMP-3, or others) (Figure 3D). Our results highlight a role of histone acetylation in ECM remodelling, that of modulating the balance between the active *versus* the latent forms of MMPs.

### TSA induces histone acetylation at the TIMP-1 and TIMP-3 gene promoters

**Figure 4:**
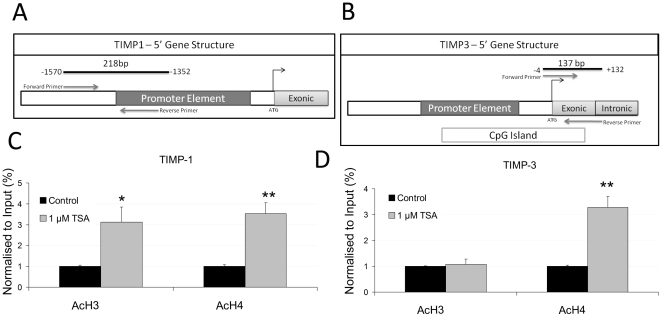
TSA induces histone acetylation at the TIMP-1 and TIMP-3 gene promoters. **A** and **B**, Schematic representation of the TIMP-1 and TIMP-3 gene promoter regions, relative position of the primers used for the Chip experiment to the translational start (ATG). Note the CpG island within the promoter element in the TIMP-3 gene. **C** and **D**, Chromatin immunoprecipitation experiments using antibodies against global H3 and H4 acetylation were carried out on the TIMP-1 (**C**) and TIMP-3 (**D**) genes in 24 hour-1 µM TSA-treated hESCs. Relative amounts of immunoprecipitated TIMP-1 and TIMP-3 promoter sequences compared to input chromatin were determined by real-time PCR. Data represent the mean of three independent experiments. Statistical analysis,* p<0.05 and ** p<0.01.

H3 and H4 histone acetylation at the TIMP-1 and TIMP-3 promoter regions was assessed by chromatin immunoprecipitation (ChIP) with specific antibodies (Figure 4A and 4B). A 24-hour treatment with 1 µM TSA in hESCs increased the amount of immunoprecipitated TIMP-1 promoter associated with acetylated H3 and H4 (3.0- and 3.5-fold, respectively) when compared to the control vehicle-treated cells (Figure 4C). Similarly, immunoprecipitated TIMP-3 promoter was associated with acetylated H4 (3.2-fold), but not with H3 (Figure 4D). Chromatin binding specificity was shown by control IgG's inability to yield an appropriate PCR product. These results demonstrate that histone acetylation at the TIMP-1 and TIMP-3 promoters after TSA treatment is associated with increased TIMP-1 and TIMP-3 mRNA expressions

### Decidualization induced the TIMP-1 and TIMP-3 expressions and histone acetylation at the gene promoter regions

**Figure 5:**
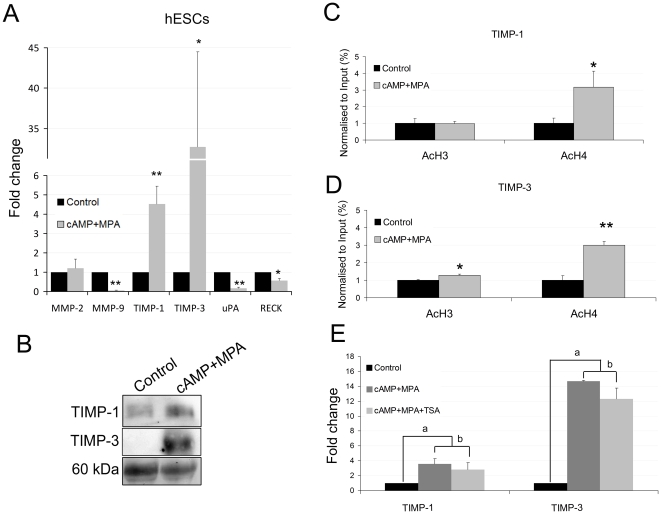
In vitro decidualized hESCs ECM modulators expression and Chip experiments at the TIMP-1 and TIMP-3 gene promoters. **A**, Quantitative PCRs for MMP-2, MMP-9, TIMP-1, TIMP-3, uPA and RECK expression levels in 5 day-cAMP+MPA-treated hESCs.. **B**, Extracellular TIMP-1 and TIMP-3 Western blot analyses of the protein extracts of 24 hour-conditioned media from hESCs treated with cAMP and MPA for 5 days. Ponceau staining of the membranes was used as a protein loading control. Only the 60 kDa band of the ponceau gel is shown. Representative image of three different analyses is shown. **C** and **D**, Chromatin immunoprecipitation experiments using antibodies against global H3 and H4 acetylation were carried out on the TIMP-1 and TIMP-3 genes in 24 hour-cAMP+MPA-treated hESCs. Relative amounts of immunoprecipitated TIMP-1 and TIMP-3 promoter sequences compared to input chromatin were determined by real-time PCR. **E**, Quantitative PCR for the TIMP-1 and TIMP-3 expression levels in 48 hour-cAMP+MPA, 1 µM TSA and cAMP+MPA+1 µM TSA-treated hESCs. Note that there is no significant difference between these two conditions at the level of the TIMP-1 and TIMP-3 gene expressions. Data represent the mean of three independent experiments. Statistical analysis,* p<0.05, ** p<0.01, a p<0.05, b p>0.05.

The decidualization process has been associated with epigenetic changes *in vivo* and *in vitro*; specifically H3 and H4 become acetylated upon decidualization [20], [21]. We postulated that histone acetylation is involved in the extensive ECM remodelling that stromal cells undergo during decidualization. *In vitro* decidualized hESCs treated with cAMP+MPA for 5 days boosted the levels of decidual marker protein prolactin (PRL) (Figure S1B), increased the mRNA levels of TIMP-1 (4.5-fold) and TIMP-3 (32.7-fold), and lowered MMP-9 (−16.6-fold), uPA (−5.8-fold) and RECK (−1.78-fold) (Figure 5A). In contrast, MMP-2 remained unaltered. At the protein level, TIMP-1 and TIMP-3 were up-regulated in the 24 hour-conditioned media from hESCs previously treated for 5 days with the decidual stimulus (Figure 5B). It is reasonable to suspect that TIMP-1 and TIMP-3 induction by the decidual stimulus could be mediated by histone acetylation. ChIP assays in 24-hour cAMP+MPA-treated hESCs revealed an increase not only in H4 acetylation (but not in H3) at the gene promoter of TIMP-1, but also in H4 and H3 acetylation at the TIMP-3 promoter (Figure 5C and 5D). Our results suggest that histone acetylation could mediate the TIMP-1 and TIMP-3 expressions during *in vitro* decidualization.

## Discussion

The formation of a functional placenta requires the extensive, although well-controlled, invasion of trophoblast cells through the stromal compartment [5]. Therefore, the mechanisms controlling this process involve both the signals that promote and limit trophoblast invasion. Trophoblast cells induce the expression of MMPs and plasminogen activators which degrade ECM components by promoting invasion, while decidual cells modulate this potential through the activation of TIMPs [32], [33], [34], [35], [36]. In addition, the motility potential of hESCs at the implantation site facilitates trophoblast invasion by anchoring the embryo onto the decidua, leading to placenta formation and successful pregnancy [27], [28]. All these processes need to be precisely controlled at a molecular level to ensure correct embryo invasion.

**Figure 6:**
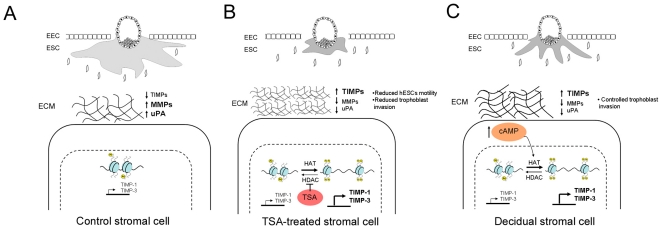
Models of the TSA-treated hESC and decidual hESCs. **A,** In control endometrial stromal cells, trophoblast invasion is massive due to low levels of TIMPs and high levels of MMPs and uPA proteases. **B,** TSA-treated hESCs inhibits HDAC and promotes histone acetylation at the promoters of TIMP-1 and TIMP-3 by increasing the transcription of these genes. In contrast, the levels and activities of MMPs and uPA decrease. These ECM modulators changes reduced the motility of hESCs and the invasion of trophoblast cells. **C**, In *in vitro* decidualized hESCs, the intracellular cAMP levels increase and promote histone acetylation at the TIMP-1 and TIMP-3 gene promoters. Moreover, the activity and level of MMPs and uPA decreases and, together, precisely control the invasion of trophoblast cells.

Treatment of mammalian cells with potent inhibitors of HDACs, such as TSA, activates or inhibits the expression of about 2% of cellular genes [37]. Our study shows that TSA-treated hESCs inhibit the expression of ECM proteases (MMP-2, MMP-9 and uPA) while activating their inhibitors (TIMP-1 and TIMP-3). Gain of TIMP-1 and TIMP-3 transcription is associated with increased histone acetylation at the promoter of these genes. This gene expression profile is associated with reduced embryo invasion in the TSA-treated hESCs. Moreover, the conditioned media from the TSA-treated hESCs diminish trophoblast cells ability to invade the ECM in a collagen barrier invasion assay, suggesting that the extracellular molecules secreted from the treated hESCs have an impact by regulating this process. The reduced trophoblast invasion described herein is in accordance with not only diminished MMPs levels and activity, but with increased extracellular TIMP-3 in the TSA-treated stromal cells (Figure 6).

In a standarized *in vitro* decidualization model, endometrial stromal cells also inhibit MMPs and produce TIMPs to antagonize the proteolytic activity of trophoblast MMPs in order to limit invasion (our results) [38]. Our experiments indicate that *in vitro* decidualized hESCs increase the TIMP-1 and TIMP-3 expressions, but decrease MMP-9 and uPA (Figure 6).

Overall, our results are in agreement with a study by Sakai et al, 2003 which proposed that histone acetylation is involved in ovarian steroid-induced decidualization [21]. Mainly, they found that not only did H3 and H4 acetylation increase in response to ovarian steroid hormones, but also a synergistic effect of HDACi TSA with E_2_ and P_4_ which enhanced the up-regulation of decidual marker genes, such as PRL and IGFBP-1. We observed H3 and H4 acetylation in the TSA-treated hESCs and in *in vitro* decidualized hESCs at the gene promoters of TIMP-1 and TIMP-3, suggesting that the post-translational modifications of histone tails could facilitate the transcriptional activation of these genes (Figure 6). Future experiments will be needed to confirm if histone acetylation mediates TIMP-1 and TIMP-3 expression during *in vitro* and *in vivo* decidualization.

We also tested the possibility of a synergistic enhancement of the cAMP+MPA-induced expression of TIMPs by TSA. The 48-hour cultures of hESCs with the decidual stimulus (cAMP+MPA) and TSA did not enhance the expression of TIMP-1 or TIMP-3 if compared to cAMP+MPA alone (Figure 5E). One possibility is that the decidual stimulus directs histone acetylation at the promoter regions of these genes, as observed in the ChIP experiments, and that the addition of TSA no longer enhances this effect. These results suggest a general mechanism for endometrial decidualization where E_2_ and P_4_ increase the levels of cAMP, thus influencing gene transcription via chromatin remodelling. Specifically, we indicate a link between ECM remodelling changes and the motility potential of decidualized stromal cells with the transcriptional control by the epigenetic histone acetylation mark (Figure 6).

HDAC inhibitors are currently being developed as cancer therapeutics, mainly because of their ability to inhibit cancer cell growth *in vitro* and *in vivo*, to induce apoptosis, and to enhance cell differentiation [39], [40]. Nonetheless, other studies propose that HDACi promotes cell invasion in some cancer cells, partly by the activation of uPA [41]. These reports confirm the need to study the effects of these drugs on each different cell type. Our results provide additional information as to how TSA affects the pattern of ECM remodelling enzymes in endometrial cells: TSA inhibits MMPs (MMP-2 and MMP-9) and uPA, while activating TIMPs (TIMP-1 and TIMP-3) in a similar manner in endometrial stromal and epithelial cells; however, this pattern is not conserved in TSA-treated foreskin fibroblast cells. In conclusion, our results highlight the role of histone acetylation during embryo invasion control by the stromal compartment of the endometrium and are important to further understand human placentation.

Some HDAC inhibitors are being studied as potential candidates for epigenetic therapies of malignant tumours [39], [42]. Therefore, the results we present herein provide information for future epigenetic therapies that target histone acetylation in reproductive diseases such as endometriosis, endometrial cancer or preeclampsia where an inadequate balance of MMPs and its inhibitors have been reported [14], [43], [44].

## Supporting Information

Figure S1 **Viability and PRL assays in TSA and cAMP+MPA treated cells.** **A,** TSA treatment at 0.1 and 1 µM does not affect the viability of hESCs. **B,** *in vitro* decidualized hESCs for 5 days with cAMP+MPA boost the Prolactin (PRL) levels in conditioned media. Data represent the mean of three independent experiments. Statistical analysis,* p<0.05.
(TIF)

## References

[1] Paria BC, Reese J, Das SK, Dey SK (2002). Deciphering the cross-talk of implantation: advances and challenges.. Science.

[2] Gellersen B, Brosens IA, Brosens JJ (2007). Decidualization of the human endometrium: mechanisms, functions, and clinical perspectives.. Semin Reprod Med.

[3] Popovici RM, Kao LC, Giudice LC (2000). Discovery of new inducible genes in in vitro decidualized human endometrial stromal cells using microarray technology.. Endocrinology.

[4] Garrido-Gomez T, Dominguez F, Lopez JA, Camafeita E, Quinonero A (2010). Modeling Human Endometrial Decidualization from the Interaction between Proteome and Secretome.. J Clin Endocrinol Metab.

[5] van den Brule F, Berndt S, Simon N, Coulon C, Le Goarant J (2005). Trophoblast invasion and placentation: molecular mechanisms and regulation.. Chem Immunol Allergy.

[6] Page-McCaw A, Ewald AJ, Werb Z (2007). Matrix metalloproteinases and the regulation of tissue remodelling.. Nat Rev Mol Cell Biol.

[7] Iwahashi M, Muragaki Y, Ooshima A, Yamoto M, Nakano R (1996). Alterations in distribution and composition of the extracellular matrix during decidualization of the human endometrium.. J Reprod Fertil.

[8] Rodgers WH, Matrisian LM, Giudice LC, Dsupin B, Cannon P (1994). Patterns of matrix metalloproteinase expression in cycling endometrium imply differential functions and regulation by steroid hormones.. J Clin Invest.

[9] Schatz F, Krikun G, Runic R, Wang EY, Hausknecht V (1999). Implications of decidualization-associated protease expression in implantation and menstruation.. Semin Reprod Endocrinol.

[10] Salamonsen LA, Nie G (2002). Proteases at the endometrial-trophoblast interface: their role in implantation.. Rev Endocr Metab Disord.

[11] Bischof P, Meisser A, Campana A (2001). Biochemistry and molecular biology of trophoblast invasion.. Ann N Y Acad Sci.

[12] Lala PK, Chakraborty C (2003). Factors regulating trophoblast migration and invasiveness: possible derangements contributing to pre-eclampsia and fetal injury.. Placenta.

[13] Knofler M (2010). Critical growth factors and signalling pathways controlling human trophoblast invasion.. Int J Dev Biol.

[14] Cockle JV, Gopichandran N, Walker JJ, Levene MI, Orsi NM (2007). Matrix metalloproteinases and their tissue inhibitors in preterm perinatal complications.. Reprod Sci.

[15] Lockwood CJ, Oner C, Uz YH, Kayisli UA, Huang SJ (2008). Matrix metalloproteinase 9 (MMP9) expression in preeclamptic decidua and MMP9 induction by tumor necrosis factor alpha and interleukin 1 beta in human first trimester decidual cells.. Biol Reprod.

[16] Bourboulia D, Stetler-Stevenson WG (2010). Matrix metalloproteinases (MMPs) and tissue inhibitors of metalloproteinases (TIMPs): Positive and negative regulators in tumor cell adhesion.. Semin Cancer Biol.

[17] Graesslin O, Cortez A, Fauvet R, Lorenzato M, Birembaut P (2006). Metalloproteinase-2, -7 and -9 and tissue inhibitor of metalloproteinase-1 and -2 expression in normal, hyperplastic and neoplastic endometrium: a clinical-pathological correlation study.. Ann Oncol.

[18] Rodriguez-Paredes M, Esteller M (2011). Cancer epigenetics reaches mainstream oncology.. Nat Med.

[19] Krusche CA, Vloet AJ, Classen-Linke I, von Rango U, Beier HM (2007). Class I histone deacetylase expression in the human cyclic endometrium and endometrial adenocarcinomas.. Hum Reprod.

[20] Munro SK, Farquhar CM, Mitchell MD, Ponnampalam AP (2010). Epigenetic regulation of endometrium during the menstrual cycle.. Mol Hum Reprod.

[21] Sakai N, Maruyama T, Sakurai R, Masuda H, Yamamoto Y (2003). Involvement of histone acetylation in ovarian steroid-induced decidualization of human endometrial stromal cells.. J Biol Chem.

[22] Simon C, Piquette GN, Frances A, el-Danasouri I, Irwin JC (1994). The effect of interleukin-1 beta (IL-1 beta) on the regulation of IL-1 receptor type I messenger ribonucleic acid and protein levels in cultured human endometrial stromal and glandular cells.. J Clin Endocrinol Metab.

[23] Tang B, Guller S, Gurpide E (1993). Cyclic adenosine 3′,5′-monophosphate induces prolactin expression in stromal cells isolated from human proliferative endometrium.. Endocrinology.

[24] Hannan NJ, Paiva P, Dimitriadis E, Salamonsen LA (2010). Models for study of human embryo implantation: choice of cell lines?. Biol Reprod.

[25] Troeberg L, Nagase H (2004). Zymography of metalloproteinases.. Curr Protoc Protein Sci Chapter.

[26] Gellersen B, Reimann K, Samalecos A, Aupers S, Bamberger AM (2010). Invasiveness of human endometrial stromal cells is promoted by decidualization and by trophoblast-derived signals.. Hum Reprod.

[27] Grewal S, Carver J, Ridley AJ, Mardon HJ (2010). Human endometrial stromal cell rho GTPases have opposing roles in regulating focal adhesion turnover and embryo invasion in vitro.. Biol Reprod.

[28] Grewal S, Carver JG, Ridley AJ, Mardon HJ (2008). Implantation of the human embryo requires Rac1-dependent endometrial stromal cell migration.. Proc Natl Acad Sci U S A.

[29] Carver J, Martin K, Spyropoulou I, Barlow D, Sargent I (2003). An in-vitro model for stromal invasion during implantation of the human blastocyst.. Hum Reprod.

[30] Andreasen PA, Kjoller L, Christensen L, Duffy MJ (1997). The urokinase-type plasminogen activator system in cancer metastasis: a review.. Int J Cancer.

[31] Takahashi C, Sheng Z, Horan TP, Kitayama H, Maki M (1998). Regulation of matrix metalloproteinase-9 and inhibition of tumor invasion by the membrane-anchored glycoprotein RECK.. Proc Natl Acad Sci U S A.

[32] Strickland S, Richards WG (1992). Invasion of the trophoblasts.. Cell.

[33] Shimonovitz S, Hurwitz A, Dushnik M, Anteby E, Geva-Eldar T (1994). Developmental regulation of the expression of 72 and 92 kd type IV collagenases in human trophoblasts: a possible mechanism for control of trophoblast invasion.. Am J Obstet Gynecol.

[34] Zhang J, Salamonsen LA (1997). Tissue inhibitor of metalloproteinases (TIMP)-1, -2 and -3 in human endometrium during the menstrual cycle.. Mol Hum Reprod.

[35] Anacker J, Segerer SE, Hagemann C, Feix S, Kapp M (2011). Human decidua and invasive trophoblasts are rich sources of nearly all human matrix metalloproteinases.. Mol Hum Reprod.

[36] Librach CL, Feigenbaum SL, Bass KE, Cui TY, Verastas N (1994). Interleukin-1 beta regulates human cytotrophoblast metalloproteinase activity and invasion in vitro.. J Biol Chem.

[37] Van Lint C, Emiliani S, Verdin E (1996). The expression of a small fraction of cellular genes is changed in response to histone hyperacetylation.. Gene Expr.

[38] Nuttall RK, Kennedy TG (1999). Gelatinases A and B and tissue inhibitors of metalloproteinases 1, 2, and 3 during in vivo and in vitro decidualization of rat endometrial stromal cells.. Biol Reprod.

[39] Johnstone RW (2002). Histone-deacetylase inhibitors: novel drugs for the treatment of cancer.. Nat Rev Drug Discov.

[40] Uchida H, Maruyama T, Nagashima T, Asada H, Yoshimura Y (2005). Histone deacetylase inhibitors induce differentiation of human endometrial adenocarcinoma cells through up-regulation of glycodelin.. Endocrinology.

[41] Pulukuri SM, Gorantla B, Rao JS (2007). Inhibition of histone deacetylase activity promotes invasion of human cancer cells through activation of urokinase plasminogen activator.. J Biol Chem.

[42] Ganesan A, Nolan L, Crabb SJ, Packham G (2009). Epigenetic therapy: histone acetylation, DNA methylation and anti-cancer drug discovery.. Curr Cancer Drug Targets.

[43] Osteen KG, Yeaman GR, Bruner-Tran KL (2003). Matrix metalloproteinases and endometriosis.. Semin Reprod Med.

[44] Di Nezza LA, Misajon A, Zhang J, Jobling T, Quinn MA (2002). Presence of active gelatinases in endometrial carcinoma and correlation of matrix metalloproteinase expression with increasing tumor grade and invasion.. Cancer.

