# Supplementary figures and images for: Inhibition of Histone Deacetylase Activity in Human Endometrial Stromal Cells Promotes Extracellular Matrix Remodelling and Limits Embryo Invasion
Source: PLoS One. 2012 Jan 26;7(1):e30508. doi: 10.1371/journal.pone.0030508 (PMC3266920; doi:10.1371/journal.pone.0030508)

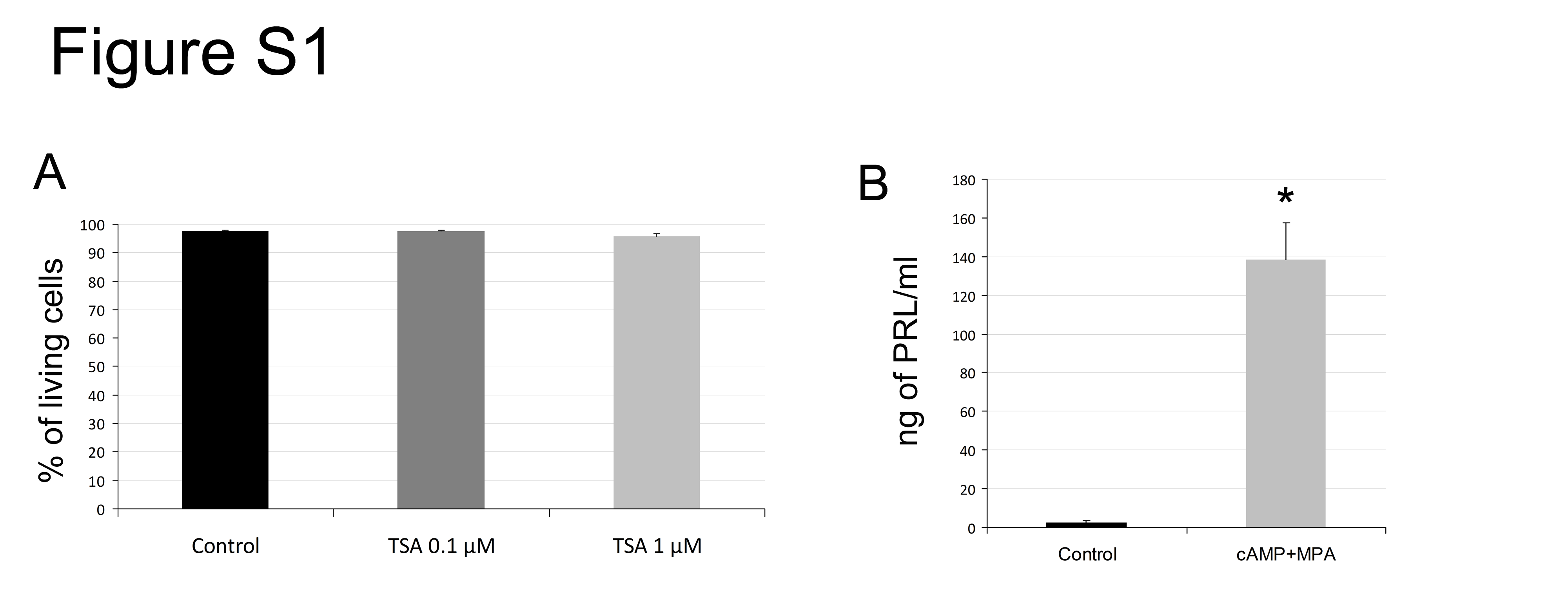

Supplement: Figure S1 — Viability and PRL assays in TSA and cAMP+MPA treated cells. A, TSA treatment at 0.1 and 1 µM does not affect the viability of hESCs. B, in vitro decidualized hESCs for 5 days with cAMP+MPA boost the Prolactin (PRL) levels in conditioned media. Data represent the mean of three independent experiments. Statistical analysis,* p<0.05. (TIF) [file pone.0030508.s001.tif]
